# Gene expression and DNA methylation analyses suggest that two immune related genes are prognostic factors of colorectal cancer

**DOI:** 10.1186/s12920-021-00966-3

**Published:** 2021-04-28

**Authors:** Xiao-Liang Xing, Zhi-Yong Yao, Chaoqun Xing, Zhi Huang, Jing Peng, Yuan-Wu Liu

**Affiliations:** 1 Xiangya Hospital, Central South University, Changsha, 410078 Hunan People’s Republic of China; 2 Hunan University of Medicine, Huaihua, 418000 Hunan People’s Republic of China; 3 Beijing Advanced Innovation Center for Food Nutrition and Human Health, China Agricultural University, Beijing, 100193 People’s Republic of China

**Keywords:** Colorectal cancer, Immune-related differentially expressed genes, Methylation, Risk model, Tumor-immune microenvironment

## Abstract

**Background:**

Colorectal cancer (CRC) is the second most prevalent cancer, as it accounts for approximately 10% of all annually diagnosed cancers. Studies have indicated that DNA methylation is involved in cancer genesis. The purpose of this study was to investigate the relationships among DNA methylation, gene expression and the tumor-immune microenvironment of CRC, and finally, to identify potential key genes related to immune cell infiltration in CRC.

**Methods:**

In the present study, we used the ChAMP and DESeq2 packages, correlation analyses, and Cox regression analyses to identify immune-related differentially expressed genes (IR-DEGs) that were correlated with aberrant methylation and to construct a risk assessment model.

**Results:**

Finally, we found that *HSPA1A* expression and *CCRL2* expression were positively and negatively associated with the risk score of CRC, respectively. Patients in the high-risk group were more positively correlated with some types of tumor-infiltrating immune cells, whereas they were negatively correlated with other tumor-infiltrating immune cells. After the patients were regrouped according to the median risk score, we could more effectively distinguish them based on survival outcome, clinicopathological characteristics, specific tumor-immune infiltration status and highly expressed immune-related biomarkers.

**Conclusion:**

This study suggested that the risk assessment model constructed by pairing immune-related differentially expressed genes correlated with aberrant DNA methylation could predict the outcome of CRC patients and might help to identify those patients who could benefit from antitumor immunotherapy.

**Supplementary Information:**

The online version contains supplementary material available at 10.1186/s12920-021-00966-3.

## Introduction

Colorectal cancer (CRC) is the second most prevalent cancer, as it accounted for approximately 10% of all diagnosed cancers worldwide in 2018 [1]. Benefits from early cancer screening programs, improved surgical techniques and the availability of more effective immune therapies for advanced-stage disease have resulted in a progressive decrease in the mortality of CRC in recent decades [2, 3]. However, a recent study indicated that the 5-year survival rate of CRC patients is relatively low, especially after surgical removal of tumors for distant stage disease (stage IV) [4]. Increasing evidence suggests that cancer malignancy depends not only on tumor cells but also on the tumor microenvironment, which includes immune cells, inflammatory mediators and extracellular matrix molecules [5]. Tumor-infiltrating immune cells are considered valuable nontumor cells in the diagnosis and prognosis of cancers [6–8]. Previous studies have demonstrated that immune cells and immune factors that constitute the tumor-immune microenvironment participate not only in antitumor immunity but also in antitumor initiation and progression [9, 10]. In recent years, signatures focusing on tumor-immune infiltration have demonstrated high value in the diagnosis, evaluation and treatment of many cancers [11–13]. Cancer immunotherapy has demonstrated promising advantages in the treatment efficiency and long-term survival of patients [14]. Therefore, it is crucial and necessary to investigate the tumor-immune microenvironment of CRC.

Epigenetics was originally described by Conrad H. Waddingtone in 1942. It is well accepted that cancer stem cell genesis is initiated as a result of the progressive accumulation of genetic and epigenetic alterations [15–17]. Aberrant epigenetic alterations could inactivate the expression of tumor suppressor genes and activate the expression of oncogenes, which might ultimately lead to cancer genesis. DNA methylation is an important epigenetic modification in eukaryotic cells. In this study, we aimed to develop signatures by integrating the analysis of DNA methylation, gene expression and the tumor-immune microenvironment of CRC. Finally, we found that *HSPA1A* expression and *CCRL2* expression were positively and negatively associated with the risk score of CRC, respectively. Patients in the high-risk group were more positively and negatively correlated with several types of tumor-infiltrating immune cells. Our study suggested that the risk assessment model constructed by pairing immune-related differentially expressed genes correlated with aberrant DNA methylation could predict the potential outcome of CRC patients and help to identify those patients who might benefit from antitumor immunotherapy.

## Materials and methods

### Data source and data processing

The mRNA expression data (HTSeq counts), DNA methylation data (Illumina Human Methylation 450) and the corresponding clinical information of CRC patients (predominantly those with COAD and READ) were obtained from The Cancer Genome Atlas (TCGA) (https://portal.gdc.cancer.gov/projects/TCGA-COAD. https://portal.gdc.cancer.gov/projects/TCGA-READ). In all, 497 (41 controls vs 456 cancers) and 176 (10 controls vs 166 cancers) COAD and READ samples, respectively, were included in the gene expression profiling, while 334 (38 controls vs 296 cancers) and 105 samples (7 controls vs 98 cancers) were included in the methylation analysis for COAD and READ, respectively. After excluding those patients without RNA-seq data and methylation data, 620 CRC samples with RNA-seq data, 620 CRC samples with methylation data and 616 CRC samples with RNA-seq and methylation data were included (Table 1).

**Table 1:** CRC clinical characteristics

CRC clinical variables	CRC clinical values		
	RNA-seq data only	Methylation data only	Both RNA-seq data and methylation data
Age (mean ± sem)	66.27 ± 0.51	66.21 ± 0.51	66.22 ± 0.52
Sex (male/female)	330/290	329/291	327/289
Vital (alive/dead)	490/130	491/129	487/129
Pathologic M (M0/M1/MX/NA)	459/88/64/9	458/89/64/9	455/88/64/9
Pathologic N (N0/N1/N2/NX/NA)	351/150/116/2/1	351/149/117/2/1	348/149/116/2/1
Pathologic T (T1/T2/T3/T4/TX)	20/105/423/70/2	20/105/423/70/1/1	20/104/420/70/1/1
Pathologic stage (I/II/III/IV/NA)	105/227/179/89/20	104/228/178/90/20	104/225/178/89/20

The differential expression analysis of the mRNA profile data (raw counts) was analyzed using the DESeq2 package in R (3.6.1) software. The criteria were set as follows: adj. *P* < 0.05, |logFC|≥ 0.5 and baseMean > 50 [18–20]. The methylation levels were analyzed using the ChAMP package in R (3.6.1) software [21]. In addition, the criteria were set as follows: adj. *P* < 0.05, delta Beta value ≥ 0.2. A list of identified immune-related genes was downloaded from the ImmPort database (http://www.immport.org) [22] and was used to screen IR-DEGs using a coexpression strategy. The extent of immune cell infiltration was obtained from the Tumor IMmune Estimation Resource (TIMER), which estimates the immune cell infiltrates in patients from the TCGA database (https://cistrome.shinyapps.io/timer/).

### Correlation analysis

Spearman correlation analysis was performed for the IR-DEGs and the DMPs to identify their relationship. The specific criteria for the correlation analysis were set as a *p* value < 0.05 and |R|> 0.3. Spearman correlation analysis was performed to determine the risk score and the infiltration in CRC patients with high risk scores. The specific criteria for the correlation analysis were set as a *p* value < 0.05.

### Survival analysis

The data required for the survival analyses (such as IR-DEG expression and the risk score) were used to divide the patients into high and low expression groups according to the median value of gene expression. We used RegParallel and survival packages in R (3.6.1) to perform univariate and multivariate Cox regression analyses for a single factor and multiple factors, respectively. The factors that were verified by univariate Cox regression analysis were entered into the multivariate Cox regression analysis. IBM SPSS statistics 22 was applied for the Kaplan–Meier analysis.

### Risk model construction

After the multivariate Cox regression analysis, we constructed specific prognostic models according to previous reports [23, 24]. $\mathrm{Risk Score}=\mathrm{ExpRNA}1*\mathrm{\beta RNA}1+\mathrm{ExpRNA}2*\mathrm{\beta RNA}2+$… + $\mathrm{ExpRNAn}*\mathrm{\beta RNAn}$. Patients whose risk scores were higher than the median value were placed into the high-risk group, and patients whose risk scores were lower than the median value were placed into the low-risk group. We performed a survival analysis to determine the relationship of this model with the survival rate of CRC patients. Then, we constructed time-dependent receiver operating characteristic (ROC) curves for 3 years, 5 years and 10 years and estimated their utility as a prognostic model for predicting survival status. A repeated-measure ANOVA followed by Bonferroni post hoc tests or unpaired two-tailed Student’s t test was used as indicated. All statistical analyses were performed using GraphPad Prism 6.01.

## Results

### Identification of immune-related DEGs associated with DMPs

**Fig. 1:**
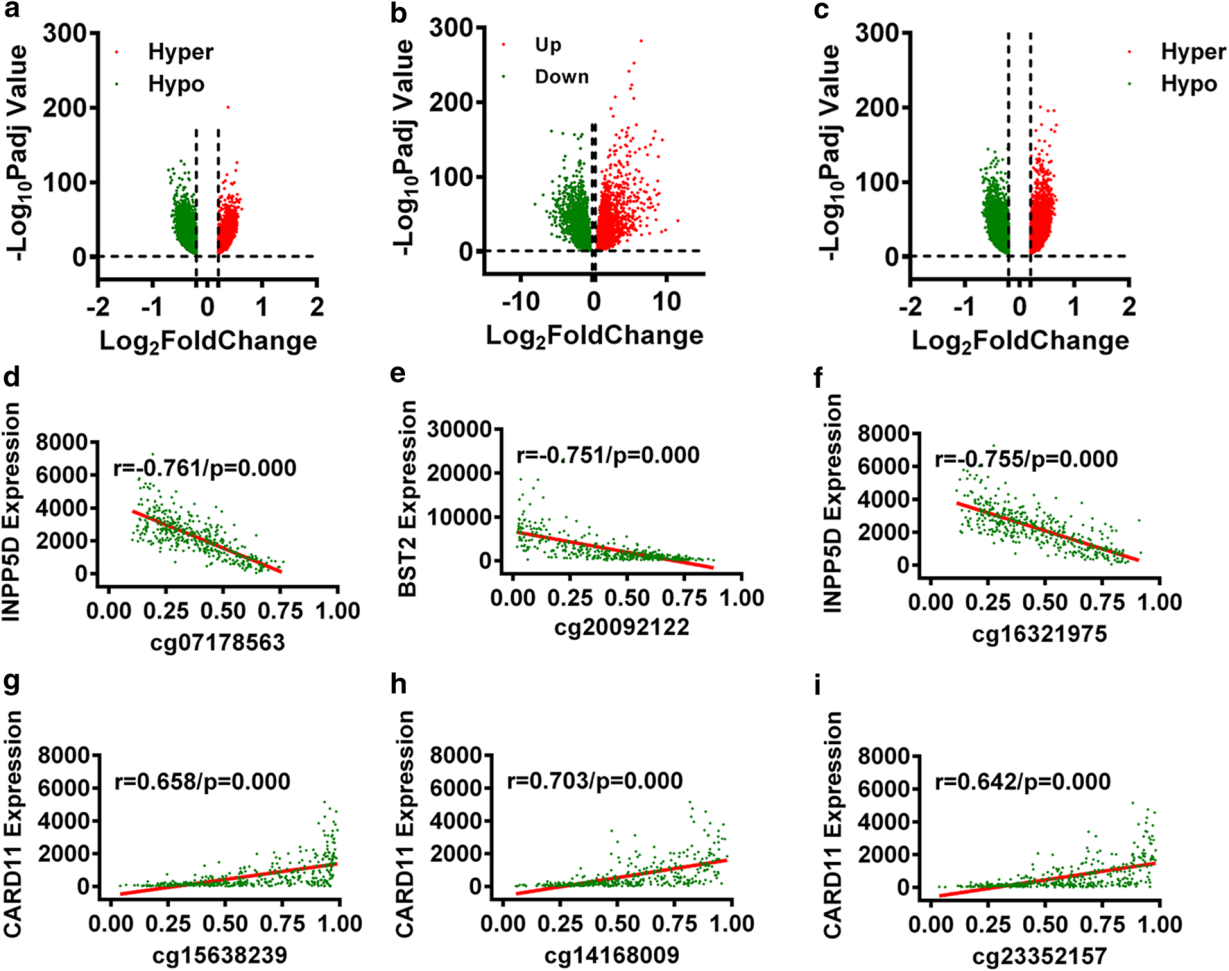
Identification of immune-related DEGs correlated with DMPs. **a** Volcano plot of DMPs for CRC. **b** Volcano plot of DMPs in promoter regions for CRC. **c** Volcano plot of DEGs for CRC. **d**–**f** The top three pairs of DMPs and IR-DEGs with negative correlations. **g**–**i** The top three pairs of DMPs and IR-DEGs with positive correlations

Based on a differential methylation analysis using ChAMP, we screened 40,936 DMPs (21,998 hypermethylated and 18,938 hypomethylated) out of 111,336 methylation probes (Fig. 1a). In the present study, we only considered the DMPs located in promoter regions (5′UTR, TSS1500 and TSS200) for further analysis (1320 hypermethylated and 10,651 hypomethylated genes that further affected 4180 genes) (Fig. 1b). Similarly, we identified 7616 DEGs (4111 upregulated and 3505 downregulated) through differential expression analysis using DESeq2 (Fig. 1c). Of these DEGs, 625 were immune-related DEGs (IR-DEGs) (Additional file 1: Table S1).

To determine the relationship of those 625 IR-DEGs and 11,971 DMPs, we performed a Spearman correlation analysis and obtained 137 pairs of DMPs-IR-DEGs. In all, 101 negative correlation pairs involving 101 DMPs and 40 IR-DEGs and 36 positive correlation pairs involving 36 DMPs and 21 IR-DEGs were found (Additional file 1: Table S2). The top three pairs of DMPs and IR-DEGs with negative and positive correlations are displayed in Fig. 1d–i. The distributions of the three DMPs are also shown in Additional file 1: Figure S1.

### Risk assessment model construction and clinical evaluation

**Fig. 2:**
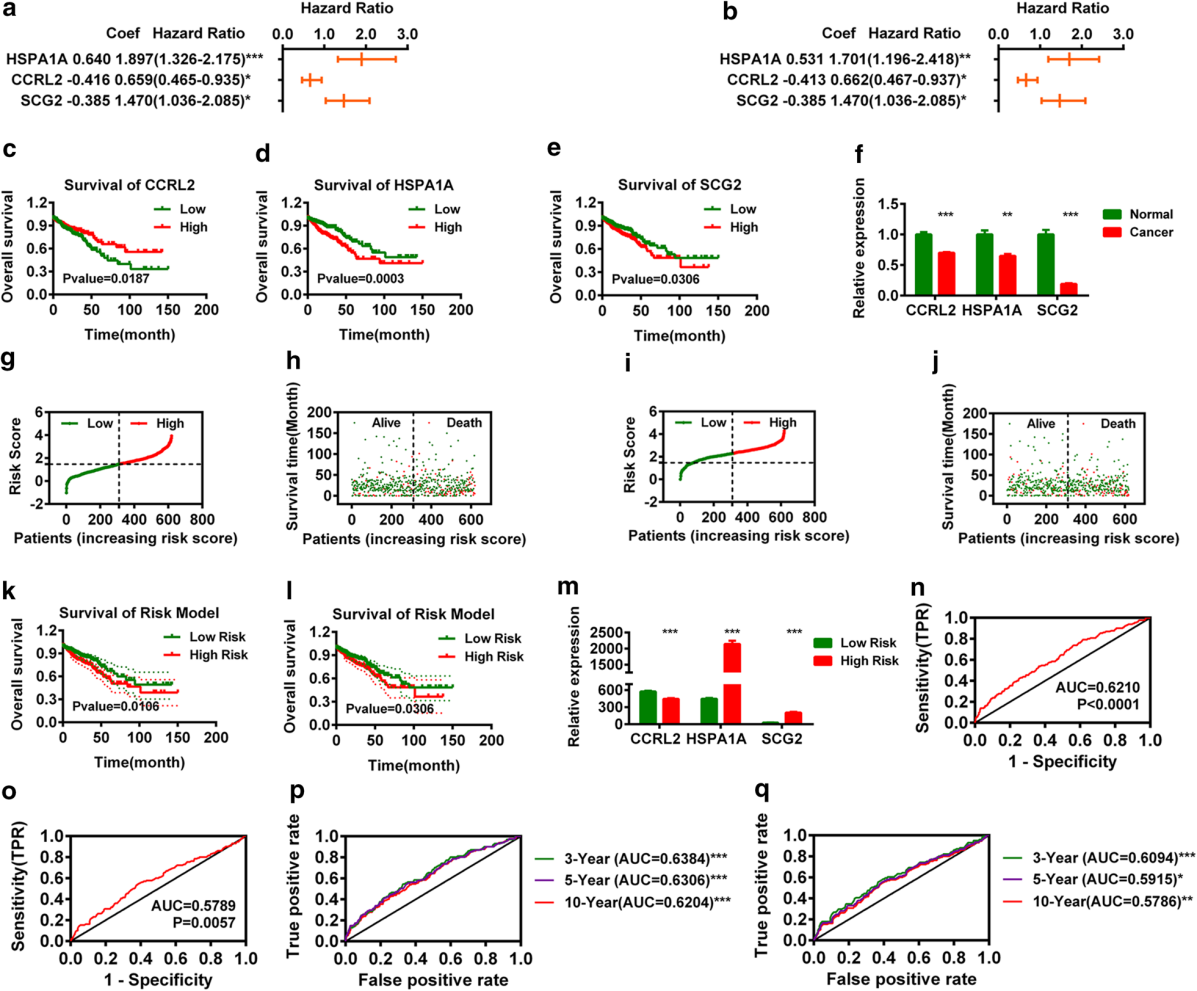
Construction of a Risk Assessment Model based on *CCRL2*, *HSPA1A* and *SCG2*. Univariate Cox (**a**) and multivariate Cox (**b**) regression analyses for *CCRL2*, *HSPA1A* and *SCG2*. **c**–**e** Overall survival analysis for *CCRL2*, *HSPA1A* and *SCG2* by univariate Cox regression analysis. **f** The normalized expression of *CCRL2*, *HSPA1A* and *SCG2* in CRC. Risk scores (**g**) and survival outcomes (**h**) of each case of CRC based on *CCRL2* and *HSPA1A*. Risk scores (**i**) and survival outcomes (**j**) of each case of CRC based on *CCRL2* and *HSPA1A*. Overall survival analysis of the risk model constructed based on 2 IR-DEGs (*CCRL2* and *HSPA1A*) (**k**) and 1 DEG (*SCG2*) (**l**). **m** The expression of *CCRL2*, *HSPA1A* and SCG2 in different groups of CRC. The ROC curve of the risk model based on 2 IR-DEGs (*CCRL2* and *HSPA1A*) (**n**) and 1 DEG (*SCG2*) (**o**). The AUC values of the 3-year, 5-year and 10-year survival based on 2 IR-DEGs (*CCRL2* and *HSP1A1*) (**p**) and 1 DEG (*SCG2*) (**q**)

For those 40 and 21 IR-DEGs, we first performed univariate and multivariate Cox analyses and found that 2 IR-DEGs (*CCRL2* and *HSPA1A*) out of 40 negatively correlated IR-DEGs and 1 IR-DEG (*SCG2*) out of 21 positively correlated IR-DEGs were correlated with the overall survival (OS) of CRC patients (Fig. 2a–e). The expression of *CCRL2*, *HSPA1A* and *SCG2* was significantly decreased in CRC (Fig. 2f).

We constructed two risk assessment models based on those IR-DEGs (*CCRL2*, *HSPA1A* and *SCG2*). The risk scores and survival times of each patient are shown in Fig. 2g, h and Fig. 2i, j. The survival analysis of both models indicated that the low-risk group of CRC patients (n = 310) displayed a better OS rate than the high-risk group of CRC patients (n = 309) (Fig. 2k, l). Next, we calculated the areas under the curve (AUCs) for each ROC curve of the 3 IR-DEGs (*CCRL2, HSPA1A* and *SCG2*) and drew the curved line (Fig. 2n, o). We also plotted the 3-year, 5-year and 10-year ROC curves, which indicated that the AUC values were 0.6384/0.6094, 0.6306/0.5915, and 0.6204/0.5786, respectively (Fig. 2p, q). Since the risk model based on *CCRL2* and *HSPA1A* was more accurate than the risk model based on *SCG2*, the risk model based on *CCRL2* and *HSPA1A* was selected for subsequent analysis.

**Fig. 3:**
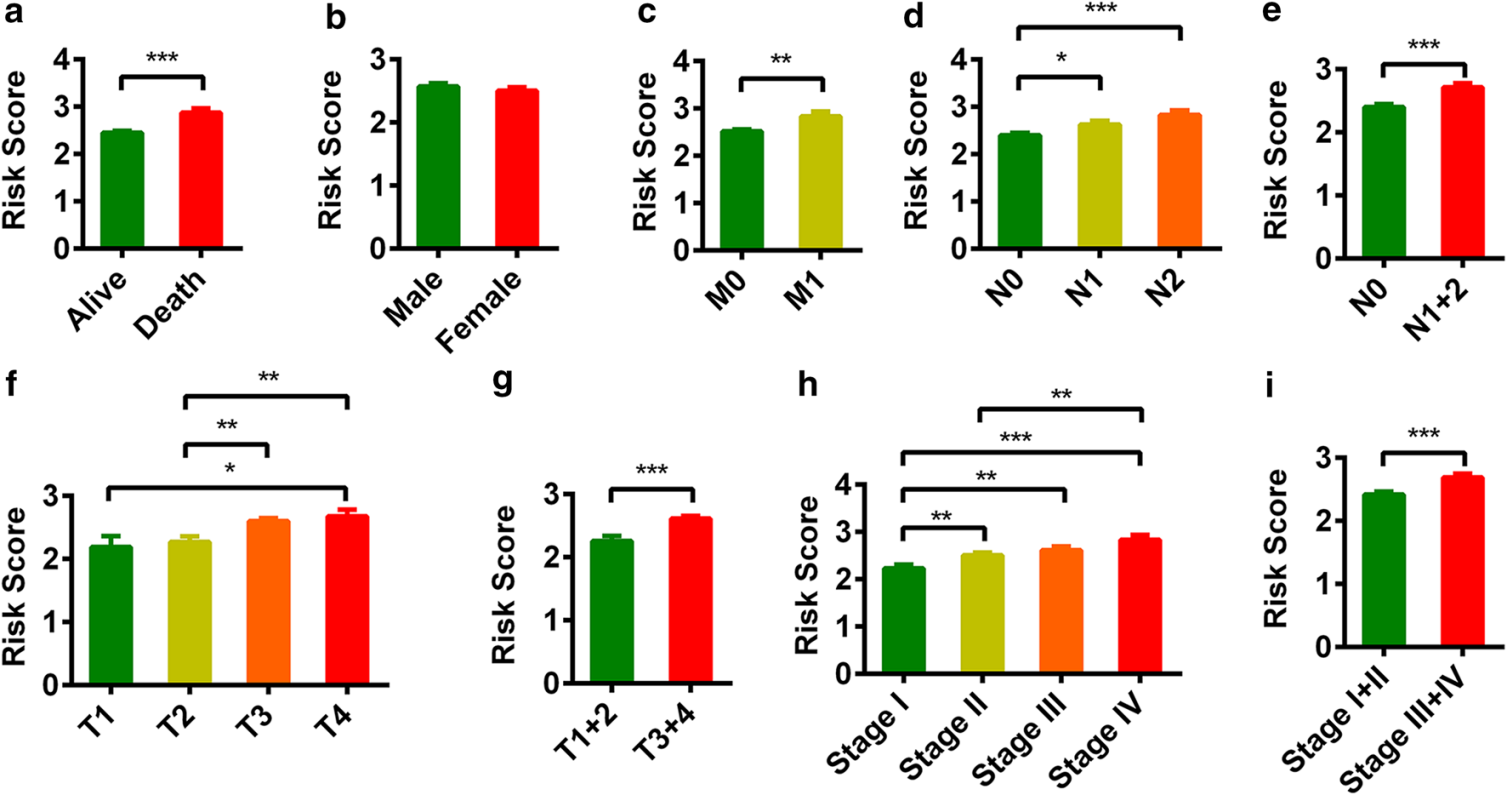
Clinical evaluations with the risk model. The histogram shows that vital status (**a**), pathologic M (**c**), pathologic N (**d**, **e**), pathologic T (**f**, **g**) and pathologic stage (**h**, **i**) were significantly associated with the risk score. Gender (**b**) was not associated with the risk score

Subsequently, we performed a series of analyses to investigate the relationship between the risk score and the clinical characteristics. We found that vital status, pathologic T stage, pathologic N stage, pathologic M stage and pathologic stage were significantly related to risk (Fig. 3a–i).

### Estimation of tumor-infiltrating immune cells and immune molecules using the risk assessment model

Since DEGs and immune-related genes were initially connected, we investigated whether the risk score was related to the three signatures (*CCRL2*, *HSPA1A* and *SCG2*) and found that *CCRL2* was negatively correlated with the risk scores and that *HSPA1A* and *SCG2* were positively correlated with the risk score (Fig. 2m).

In addition, we also investigated whether the risk model was related to the tumor-immune microenvironment. We determined the relationship between immune infiltration status and cancer and found that 93 immune cells and immune molecules were significantly different between the control and cancer samples (Additional file 1: Table S3).

**Fig. 4:**
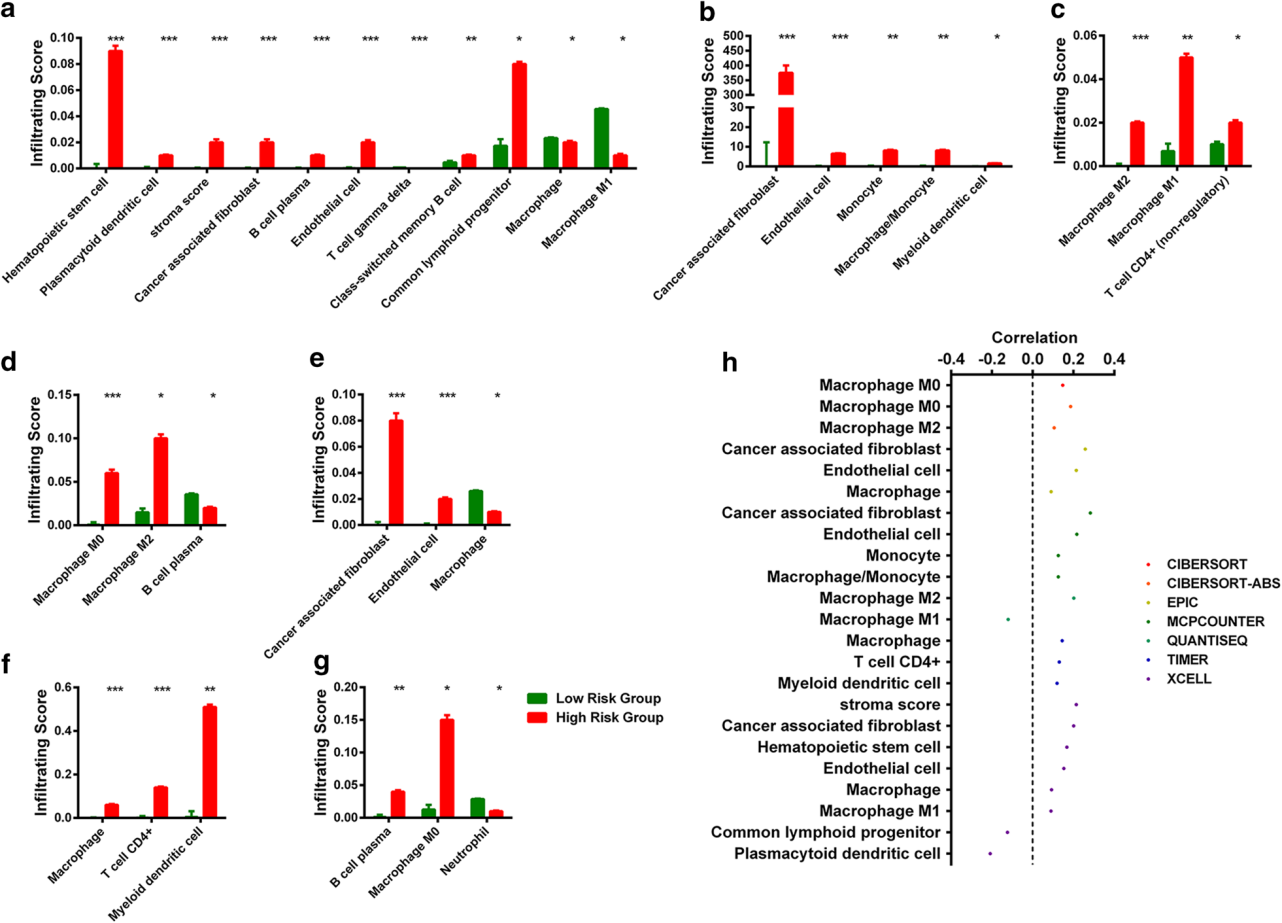
Estimation of tumor-infiltrating immune cells and immune molecules using the risk assessment model. Tumor-Infiltrating Immune Cells (**a** XELL, **b** MCPCOUNTER, **c** QUANTISEQ, **d** CIBERSORT-ABS, **e** EPIC, **f** TIMER, **g** CIBERSORT) was significantly associated with the risk score, **h** patients in the high-risk group were more positively associated with tumor-infiltrating immune cells and immune molecules, whereas they were negatively associated with other tumor-infiltrating immune cells and immune molecules

By further analysis, we found that 31 immune cells and immune molecules differed between the high-risk and low-risk groups (Fig. 4a–g). A detailed Spearman correlation analysis was conducted, and the results demonstrated that 20 immune cells and immune molecules were positively correlated with a high risk score and that 3 immune cells and immune molecules were negatively correlated with a high risk score (Fig. 4h).

## Discussion

The development of cancer genesis is controlled by complex biological processes that are the result of genetic and epigenetic abnormalities and the interaction between cancer cells and their microenvironments, which include immune cells and immune factors. Cancer immunotherapy has demonstrated promising advantages in the treatment efficiency and long-term survival of cancer patients [25]. For example, Chen et al. reported that CRC patients with different subtypes exhibit different survival outcomes and immune infiltration patterns [26]. Immune checkpoint inhibitor therapy is more effective in low-risk CRC subtypes. Therefore, an understanding of the immune infiltration in cancer patients is crucial for immunotherapy. Changes in DNA methylation, a major epigenetic modification, are a common phenomenon in the development of several cancers, such as colorectal cancer, breast cancer and pancreatic cancer [27, 28]. Aberrant methylation can alter target gene expression. In this study, we were inspired by related strategies to use immune-related genes and abnormal methylation data to construct rational risk models.

In this work, we screened 40 IR-DEGs by integrated analysis and identified two IR-DEGs (*HSPA1A* and *CCRL2*) that were correlated with the OS of CRC patients according to the Cox regression analysis. HSPA1A (heat shock protein family A member 1A) is a cytosolic molecular chaperone that is essential for cellular homeostasis. Previous studies demonstrated that HSPA1A has a powerful immune function. It can activate the classical complement pathway, participate in the processing and presentation of exogenous antigens and show immune reactivity to endogenous heat shock proteins [29–31]. In 2020, Andrada et al. reported that HSPA1A was underexpressed in primary and metastatic epithelial ovarian cancer and negatively correlated with the risk of mortality in ovarian cancer patients [32]. In this study, we found that the expression of *HSPA1A* was decreased significantly in CRC and that CRC patients with low *HSPA1A* expression exhibited better overall survival. In CRC patients with high risk scores, the *HSPA1A* expression level was significantly increased. CRC patients with high risk scores were more positively associated with tumor-infiltrating immune cells, such as cancer-associated fibroblasts, endothelial cells, M0/1/2 macrophages, and CD4+ T cells. Interestingly, Zhang et al. also found that the lncRNA HOTAIR facilitates the expression of *HSPA1A* by sequestering miR-449b-5p posttranscriptionally and thereby endows breast cancer with radiation resistance [33]. These results suggested that HSPA1A may be a potential target for breast cancer radiotherapy. Considering our results, we proposed that HSPA1A could serve as a potential target for CRC therapy.

CCRL2 (C–C chemokine receptor-like 2) is a nonsignaling receptor for chemerin. CCRL2 binds chemerin, a protein that promotes chemotaxis of leukocytes, including macrophages and natural killer (NK) cells [34]. Prete et al. (2019) found that *CCRL2* expression was increased significantly in biopsies of human lung adenocarcinoma and that it was positively correlated with clinical outcome [34]. Additionally, they also found that deletion of the *CCRL2* gene promoted tumor progression [34]. Wang et al. reported that *CCRL2* expression was downregulated in highly invasive human breast cancer cells. They also reported that stable overexpression of *CCRL2* in highly invasive cell lines attenuated chemotaxis and invasion stimulated by its ligand CCL2 [35]. These results provide evidence for the crucial role of CCRL2 in shaping an antitumor-immune response. In the present study, we found that the expression of *CCRL2* in CRC patients and CRC patients with high risk scores was significantly decreased compared with that in normal patients and CRC patients with low risk scores.

In conclusion, this study demonstrated that a novel signature constructed by pairing IR-DEGs (*HSPA1A* and *CCRL2*) could predict the outcomes of patients with CRC and may help to distinguish those patients who might benefit from antitumor immunotherapy. However, this study had some limitations; for example, the use of multiple sources of clinical datasets would be beneficial for cross validation. Therefore, we plan to collect clinical samples and expand the sample size for further verification in future studies.

## Supplementary Information

**Additional file 1.** **Supplementary table 1.** Immune-related DEGs for CRC. **Supplementary table 2.** The screened pairs of DMPs and IR-DEGs by Spearman correlation analysis. **Supplementary table 3.** Tumor-infiltrating immune cells correlated with the risk between normal and cancer.

## Data Availability

The datasets analyzed during the current study are available in TCGA repository (https://portal.gdc.cancer.gov/projects/TCGA-COAD. https://portal.gdc.cancer.gov/projects/TCGA-READ), under the accession code: Colon adenocarcinoma (COAD), Rectum adenocarcinoma (READ).

## Abbreviations

CRC: Colorectal cancer
COAD: Colon adenocarcinoma
READ: Rectum adenocarcinoma
IR: Immune related
DEGs: Differentially expressed genes
DMP: Differential methylation probe
