# Supplementary material for: Gene expression and DNA methylation analyses suggest that two immune related genes are prognostic factors of colorectal cancer
Source: BMC Med Genomics. 2021 Apr 28;14:116. doi: 10.1186/s12920-021-00966-3 (PMC8080337; doi:10.1186/s12920-021-00966-3)
Supplement: Supplementary file 1 — Additional file 1. Supplementary table 1. Immune-related DEGs for CRC. Supplementary table 2. The screened pairs of DMPs and IR-DEGs by Spearman correlation analysis. Supplementary table 3. Tumor-infiltrating immune cells correlated with the risk between normal and cancer. [file 12920_2021_966_MOESM1_ESM.docx]

**Gene expression and DNA methylation analyses suggest that two immune related genes are prognostic factors of colorectal cancer**

Xiao-Liang Xing^1,2^, Zhi-Yong Yao^2^, Chaoqun Xing^2^, Zhi Huang^2^, Jing Peng^1*^, Yuan-Wu Liu^3*^

^1^Xiangya Hospital, Central South University, Changsha, 410078, Hunan, P. R. China.

^2^Hunan University of Medicine, Huaihua 418000, Hunan, P. R. China.

^3^Beijing Advanced Innovation Center for Food Nutrition and Human Health, China Agricultural University, 100193, Beijing, China.

*Correspondence: Yuan-Wu Liu, yuanwu_liu@126.com; Jing Peng, pengjing4346@163.com

**Supplementary information: 3 tables and 1 figures**

**Additional file 1: Table S1 Immune-related DEGs for CRC.**

| **Gene Name** | **baseMean** | **log2FoldChange** | **padj** |
| --- | --- | --- | --- |
| FGF19 | 122.13 | 6.57 | 0.0000 |
| PPBP | 239.97 | 5.89 | 0.0000 |
| ESM1 | 246.26 | 5.52 | 0.0000 |
| CXCL5 | 1024.42 | 5.09 | 0.0000 |
| INHBA | 1265.99 | 5.07 | 0.0000 |
| IGF2 | 30605.41 | 4.67 | 0.0000 |
| IL11 | 170.46 | 4.65 | 0.0000 |
| FABP6 | 350.39 | 4.65 | 0.0000 |
| PGC | 58.23 | 4.42 | 0.0000 |
| SSTR5 | 64.50 | 4.30 | 0.0000 |
| MUC5AC | 1447.91 | 4.20 | 0.0000 |
| AMH | 131.17 | 4.18 | 0.0000 |
| ULBP2 | 67.32 | 4.14 | 0.0000 |
| TG | 365.12 | 4.10 | 0.0000 |
| S100A2 | 332.17 | 4.09 | 0.0000 |
| SPP1 | 5340.87 | 3.81 | 0.0000 |
| PRKCG | 84.42 | 3.69 | 0.0000 |
| SERPIND1 | 57.70 | 3.59 | 0.0000 |
| STC2 | 980.26 | 3.55 | 0.0000 |
| OLR1 | 174.72 | 3.53 | 0.0000 |
| LCN15 | 1307.29 | 3.39 | 0.0000 |
| DEFA6 | 714.54 | 3.37 | 0.0000 |
| CXCL8 | 3576.09 | 3.34 | 0.0000 |
| TNFSF9 | 321.01 | 3.24 | 0.0000 |
| APLN | 341.04 | 3.24 | 0.0000 |
| AZGP1 | 2668.32 | 3.23 | 0.0000 |
| BMP7 | 1543.46 | 3.20 | 0.0000 |
| TDGF1 | 998.60 | 3.02 | 0.0000 |
| GDF15 | 6776.81 | 3.01 | 0.0000 |
| DKK1 | 65.41 | 2.96 | 0.0000 |
| CXCL3 | 1642.24 | 2.92 | 0.0000 |
| CXCL1 | 2855.85 | 2.90 | 0.0000 |
| LGR5 | 2691.09 | 2.87 | 0.0000 |
| EREG | 1961.07 | 2.86 | 0.0000 |
| IGLJ2 | 75.66 | 2.84 | 0.0000 |
| TFR2 | 171.58 | 2.84 | 0.0000 |
| NOX4 | 84.21 | 2.81 | 0.0000 |
| ANOS1 | 297.26 | 2.79 | 0.0000 |
| IL23A | 104.66 | 2.79 | 0.0000 |
| OXTR | 78.90 | 2.76 | 0.0000 |
| REG1A | 11966.11 | 2.68 | 0.0000 |
| NR0B2 | 143.41 | 2.53 | 0.0000 |
| CXCL11 | 525.32 | 2.52 | 0.0000 |
| IL1A | 91.66 | 2.52 | 0.0000 |
| SAA2 | 108.76 | 2.49 | 0.0000 |
| FGFRL1 | 3427.45 | 2.48 | 0.0000 |
| PCSK1 | 943.16 | 2.43 | 0.0000 |
| INHBB | 340.72 | 2.36 | 0.0000 |
| SLC11A1 | 321.24 | 2.36 | 0.0000 |
| CXCL2 | 916.11 | 2.33 | 0.0000 |
| LCN2 | 23810.13 | 2.33 | 0.0000 |
| S100P | 8783.53 | 2.31 | 0.0000 |
| CXCL6 | 107.93 | 2.25 | 0.0000 |
| TNFRSF12A | 2002.66 | 2.24 | 0.0000 |
| GZMB | 474.90 | 2.23 | 0.0000 |
| OSM | 207.39 | 2.22 | 0.0000 |
| LCN12 | 164.33 | 2.19 | 0.0000 |
| AGT | 1024.84 | 2.16 | 0.0000 |
| VGF | 126.32 | 2.15 | 0.0000 |
| ZC3HAV1L | 279.49 | 2.13 | 0.0000 |
| AQP9 | 183.66 | 2.12 | 0.0000 |
| LGR6 | 959.78 | 2.11 | 0.0000 |
| RBP2 | 195.19 | 2.08 | 0.0000 |
| DEFA5 | 784.41 | 2.08 | 0.0000 |
| PLAU | 2401.46 | 2.06 | 0.0000 |
| JAG2 | 1681.60 | 2.05 | 0.0000 |
| IGHG1 | 45605.24 | 2.04 | 0.0000 |
| FGF18 | 77.65 | 2.02 | 0.0000 |
| R3HDML | 64.15 | 2.00 | 0.0000 |
| STC1 | 504.52 | 1.98 | 0.0000 |
| IGHG3 | 5594.09 | 1.96 | 0.0000 |
| ULBP3 | 52.78 | 1.96 | 0.0000 |
| BMP4 | 2825.93 | 1.95 | 0.0000 |
| TNFRSF11B | 644.53 | 1.94 | 0.0000 |
| PF4 | 86.37 | 1.91 | 0.0000 |
| S100A11 | 15113.70 | 1.91 | 0.0000 |
| NMB | 249.81 | 1.90 | 0.0000 |
| S100A3 | 66.79 | 1.90 | 0.0000 |
| ROBO2 | 200.22 | 1.89 | 0.0000 |
| IFITM1 | 10560.90 | 1.88 | 0.0000 |
| CCL24 | 1342.46 | 1.86 | 0.0000 |
| PDIA2 | 70.43 | 1.85 | 0.0000 |
| LIF | 1208.07 | 1.82 | 0.0000 |
| CSF3 | 110.59 | 1.81 | 0.0000 |
| IL1RN | 677.65 | 1.79 | 0.0000 |
| AREG | 3443.67 | 1.79 | 0.0000 |
| EDNRA | 472.94 | 1.79 | 0.0000 |
| MET | 5564.31 | 1.76 | 0.0000 |
| CXCL10 | 877.11 | 1.74 | 0.0000 |
| TNFSF15 | 571.69 | 1.74 | 0.0000 |
| MMP12 | 2141.82 | 1.72 | 0.0000 |
| IGHG4 | 6475.75 | 1.68 | 0.0000 |
| TUBB3 | 107.95 | 1.65 | 0.0000 |
| UMODL1 | 50.66 | 1.64 | 0.0000 |
| CCL20 | 2143.48 | 1.63 | 0.0000 |
| TNFRSF10C | 100.04 | 1.62 | 0.0000 |
| PDF | 191.55 | 1.56 | 0.0000 |
| LTB4R | 274.70 | 1.53 | 0.0000 |
| RBP1 | 798.12 | 1.53 | 0.0000 |
| VEGFA | 6090.51 | 1.51 | 0.0000 |
| MC1R | 131.82 | 1.49 | 0.0000 |
| SAA1 | 379.71 | 1.48 | 0.0000 |
| HSPA6 | 164.48 | 1.48 | 0.0000 |
| PGF | 379.66 | 1.47 | 0.0000 |
| CMTM7 | 745.80 | 1.46 | 0.0000 |
| NOD2 | 238.05 | 1.44 | 0.0000 |
| BIRC5 | 2540.00 | 1.43 | 0.0000 |
| FGFR4 | 4407.73 | 1.43 | 0.0000 |
| GAL | 426.22 | 1.42 | 0.0000 |
| RBP4 | 625.72 | 1.40 | 0.0000 |
| WNT5A | 847.78 | 1.39 | 0.0000 |
| MIF | 2194.81 | 1.39 | 0.0000 |
| PLXNA1 | 3035.94 | 1.36 | 0.0000 |
| CD70 | 56.35 | 1.36 | 0.0000 |
| TNFSF11 | 121.31 | 1.36 | 0.0000 |
| MMP9 | 1809.72 | 1.35 | 0.0000 |
| LYZ | 18481.81 | 1.35 | 0.0000 |
| IL20RA | 1032.12 | 1.35 | 0.0000 |
| CMTM8 | 563.02 | 1.35 | 0.0000 |
| AEN | 1653.68 | 1.33 | 0.0000 |
| TNFRSF19 | 226.20 | 1.32 | 0.0000 |
| RAC3 | 188.10 | 1.29 | 0.0000 |
| TAFA5 | 159.23 | 1.28 | 0.0000 |
| TNFRSF10B | 3088.66 | 1.24 | 0.0000 |
| CRABP2 | 276.49 | 1.23 | 0.0000 |
| CDK4 | 3880.95 | 1.22 | 0.0000 |
| PLXNA3 | 2211.66 | 1.21 | 0.0000 |
| SYTL1 | 743.88 | 1.19 | 0.0000 |
| TNFSF4 | 159.30 | 1.18 | 0.0000 |
| SEMA3F | 1533.49 | 1.13 | 0.0000 |
| HSP90AB1 | 52469.99 | 1.08 | 0.0000 |
| INPP5D | 2238.42 | 1.06 | 0.0000 |
| ARG2 | 161.75 | 1.06 | 0.0000 |
| ADRM1 | 6125.32 | 1.05 | 0.0000 |
| PROCR | 1466.38 | 1.05 | 0.0000 |
| BID | 2129.63 | 1.05 | 0.0000 |
| CTLA4 | 73.05 | 1.04 | 0.0000 |
| IL1RL2 | 88.62 | 1.04 | 0.0000 |
| S100A9 | 1332.52 | 1.04 | 0.0000 |
| FPR2 | 65.52 | 1.04 | 0.0006 |
| PLCG1 | 3184.52 | 1.03 | 0.0000 |
| IL1B | 780.48 | 1.00 | 0.0000 |
| F2R | 1357.36 | 0.98 | 0.0000 |
| CD320 | 3547.76 | 0.97 | 0.0000 |
| TNFRSF4 | 139.27 | 0.97 | 0.0000 |
| CLEC11A | 427.75 | 0.97 | 0.0000 |
| FCGR3A | 1014.36 | 0.95 | 0.0000 |
| CCL3L1 | 86.16 | 0.94 | 0.0003 |
| SOCS1 | 287.14 | 0.91 | 0.0000 |
| IDO1 | 576.33 | 0.91 | 0.0013 |
| CXCL16 | 3478.05 | 0.90 | 0.0000 |
| LTB4R2 | 51.45 | 0.90 | 0.0000 |
| TRIM27 | 3076.09 | 0.89 | 0.0000 |
| SEMA5B | 55.25 | 0.89 | 0.0000 |
| LTBP2 | 1681.99 | 0.88 | 0.0000 |
| IL1RAP | 304.36 | 0.88 | 0.0000 |
| ADM2 | 421.69 | 0.87 | 0.0000 |
| RARG | 1302.98 | 0.87 | 0.0000 |
| PDGFRB | 3287.42 | 0.87 | 0.0000 |
| BST2 | 2663.21 | 0.86 | 0.0001 |
| PI3 | 3394.72 | 0.86 | 0.0027 |
| DUOX2 | 5963.60 | 0.86 | 0.0049 |
| TNFRSF10A | 739.78 | 0.85 | 0.0000 |
| PSMD14 | 3017.81 | 0.84 | 0.0000 |
| CARD11 | 623.84 | 0.83 | 0.0009 |
| IL27RA | 525.10 | 0.83 | 0.0000 |
| PLXNB1 | 4814.34 | 0.82 | 0.0000 |
| IL17RB | 956.34 | 0.81 | 0.0000 |
| PPIA | 17568.95 | 0.81 | 0.0000 |
| ACVR2B | 469.20 | 0.80 | 0.0000 |
| LMBR1 | 2064.09 | 0.79 | 0.0000 |
| NOX1 | 6664.26 | 0.78 | 0.0001 |
| MICB | 257.58 | 0.77 | 0.0000 |
| DUOX1 | 165.54 | 0.77 | 0.0000 |
| OGFR | 2645.24 | 0.77 | 0.0000 |
| IL6 | 168.85 | 0.76 | 0.0074 |
| CCL3 | 179.77 | 0.76 | 0.0004 |
| SHC2 | 455.99 | 0.75 | 0.0001 |
| CLCF1 | 213.56 | 0.75 | 0.0000 |
| SEM1 | 3314.57 | 0.74 | 0.0000 |
| RFXAP | 251.40 | 0.74 | 0.0000 |
| NR1D1 | 945.75 | 0.74 | 0.0000 |
| IL24 | 116.33 | 0.72 | 0.0035 |
| TNFRSF9 | 101.41 | 0.72 | 0.0000 |
| NR2F1 | 502.47 | 0.70 | 0.0000 |
| IL33 | 1475.94 | 0.69 | 0.0050 |
| PML | 2992.40 | 0.69 | 0.0000 |
| ARTN | 73.19 | 0.68 | 0.0000 |
| FPR1 | 243.43 | 0.68 | 0.0052 |
| FCGR3B | 120.47 | 0.68 | 0.0239 |
| MANF | 2852.72 | 0.68 | 0.0000 |
| GDF11 | 453.53 | 0.68 | 0.0000 |
| HSPA1B | 4451.23 | 0.67 | 0.0000 |
| NENF | 2150.59 | 0.66 | 0.0000 |
| SEMA4F | 320.88 | 0.66 | 0.0000 |
| CCL18 | 735.88 | 0.65 | 0.0288 |
| SLC29A3 | 561.92 | 0.65 | 0.0000 |
| MDK | 5749.69 | 0.63 | 0.0002 |
| GPI | 18670.34 | 0.63 | 0.0000 |
| MYDGF | 4587.15 | 0.63 | 0.0000 |
| HSP90AA1 | 40172.86 | 0.63 | 0.0000 |
| NR6A1 | 398.16 | 0.62 | 0.0000 |
| TRPC4AP | 5560.57 | 0.62 | 0.0000 |
| BMP8A | 51.91 | 0.61 | 0.0001 |
| CSF3R | 333.47 | 0.61 | 0.0064 |
| ELN | 1854.29 | 0.60 | 0.0015 |
| TMSB4XP8 | 269.65 | 0.60 | 0.0038 |
| CACYBP | 2841.57 | 0.60 | 0.0000 |
| IRF3 | 2365.15 | 0.60 | 0.0000 |
| MX2 | 668.58 | 0.59 | 0.0002 |
| TKFC | 1996.13 | 0.59 | 0.0000 |
| TNFRSF25 | 668.71 | 0.59 | 0.0001 |
| HDGF | 15058.39 | 0.59 | 0.0000 |
| GRK2 | 8159.16 | 0.58 | 0.0000 |
| SDC4 | 10395.85 | 0.58 | 0.0000 |
| PPP4C | 5073.96 | 0.57 | 0.0000 |
| SEMA4D | 1659.98 | 0.56 | 0.0000 |
| NFAT5 | 1460.92 | 0.56 | 0.0000 |
| PSMC2 | 3445.64 | 0.56 | 0.0000 |
| DLL4 | 970.64 | 0.55 | 0.0000 |
| CCL4 | 233.74 | 0.55 | 0.0056 |
| ICAM1 | 1392.24 | 0.55 | 0.0002 |
| UNC93B1 | 2499.19 | 0.55 | 0.0000 |
| HRAS | 1122.35 | 0.55 | 0.0000 |
| HSPA8 | 51928.59 | 0.54 | 0.0000 |
| CXCR2 | 67.64 | 0.53 | 0.0399 |
| PROC | 88.84 | 0.53 | 0.0033 |
| ELAVL1 | 3411.94 | 0.53 | 0.0000 |
| HMGB1 | 10340.34 | 0.53 | 0.0000 |
| ISG15 | 1894.78 | 0.53 | 0.0057 |
| LYN | 2260.18 | 0.52 | 0.0000 |
| NR2C1 | 751.68 | 0.52 | 0.0000 |
| SEMA7A | 392.84 | 0.52 | 0.0022 |
| IL17D | 73.94 | 0.52 | 0.0004 |
| PDGFB | 564.33 | 0.51 | 0.0000 |
| PLAUR | 2579.77 | 0.51 | 0.0001 |
| TAP1 | 8120.65 | 0.51 | 0.0002 |
| TFRC | 14409.59 | 0.50 | 0.0001 |
| LRSAM1 | 1082.14 | 0.50 | 0.0000 |
| IFNGR1 | 3534.17 | -0.50 | 0.0000 |
| SBDS | 3534.43 | -0.50 | 0.0000 |
| TAPBPL | 1868.26 | -0.50 | 0.0000 |
| RARB | 99.13 | -0.51 | 0.0007 |
| HLA-DRB1 | 8650.06 | -0.51 | 0.0077 |
| LGMN | 4772.43 | -0.51 | 0.0000 |
| TNFSF13B | 184.77 | -0.51 | 0.0073 |
| CCRL2 | 523.66 | -0.52 | 0.0000 |
| RASGRP3 | 261.76 | -0.52 | 0.0001 |
| SORT1 | 4671.18 | -0.53 | 0.0000 |
| CD72 | 96.51 | -0.53 | 0.0006 |
| HLA-E | 25224.37 | -0.53 | 0.0000 |
| HLA-H | 1558.57 | -0.53 | 0.0007 |
| PIK3R1 | 2110.99 | -0.54 | 0.0000 |
| TNC | 4542.62 | -0.54 | 0.0214 |
| RAC2 | 1468.42 | -0.54 | 0.0000 |
| TYROBP | 1056.94 | -0.55 | 0.0024 |
| TNFSF14 | 56.04 | -0.55 | 0.0026 |
| CD86 | 234.52 | -0.55 | 0.0020 |
| CRLF1 | 54.01 | -0.56 | 0.0003 |
| ACKR3 | 778.07 | -0.56 | 0.0001 |
| NRP1 | 1329.96 | -0.56 | 0.0002 |
| INSR | 2699.48 | -0.57 | 0.0000 |
| KLRD1 | 97.96 | -0.57 | 0.0143 |
| ROBO3 | 113.37 | -0.57 | 0.0008 |
| RASGRP1 | 109.92 | -0.58 | 0.0009 |
| CMTM4 | 4500.39 | -0.59 | 0.0000 |
| PGRMC2 | 2336.68 | -0.59 | 0.0000 |
| SEMA3C | 2601.16 | -0.59 | 0.0000 |
| APOD | 614.22 | -0.59 | 0.0287 |
| ERAP1 | 3403.12 | -0.59 | 0.0000 |
| OSGIN1 | 296.56 | -0.60 | 0.0000 |
| PLXNC1 | 465.44 | -0.60 | 0.0002 |
| PPARG | 2449.76 | -0.60 | 0.0000 |
| DDX58 | 644.42 | -0.61 | 0.0000 |
| PDGFRL | 85.08 | -0.62 | 0.0090 |
| CXCR6 | 167.65 | -0.62 | 0.0004 |
| HSPA1A | 1343.57 | -0.62 | 0.0018 |
| PPARA | 1933.25 | -0.63 | 0.0000 |
| HLA-DRA | 15284.02 | -0.63 | 0.0015 |
| ACVR2A | 497.96 | -0.64 | 0.0000 |
| CD8B | 95.32 | -0.64 | 0.0012 |
| NFKBIA | 3061.61 | -0.64 | 0.0000 |
| KCNH2 | 662.17 | -0.64 | 0.0013 |
| IL4R | 3087.16 | -0.66 | 0.0000 |
| PRKCA | 1545.41 | -0.66 | 0.0000 |
| PPP3CB | 1347.74 | -0.66 | 0.0000 |
| SLC40A1 | 8821.95 | -0.66 | 0.0000 |
| CCL2 | 506.49 | -0.67 | 0.0004 |
| C3AR1 | 312.77 | -0.67 | 0.0002 |
| IL18R1 | 107.24 | -0.67 | 0.0000 |
| BMPR1A | 931.21 | -0.68 | 0.0000 |
| TRBC2 | 427.15 | -0.68 | 0.0001 |
| IL15 | 160.75 | -0.69 | 0.0000 |
| APOBEC3C | 1200.39 | -0.69 | 0.0000 |
| C3 | 7239.98 | -0.69 | 0.0038 |
| NCK1 | 904.96 | -0.70 | 0.0000 |
| IL3RA | 332.26 | -0.71 | 0.0000 |
| HLA-G | 319.60 | -0.72 | 0.0003 |
| CCR5 | 185.83 | -0.72 | 0.0000 |
| WFDC2 | 2006.99 | -0.72 | 0.0142 |
| KRAS | 2000.97 | -0.73 | 0.0000 |
| HLA-DOA | 528.25 | -0.73 | 0.0010 |
| PLXNB3 | 123.77 | -0.73 | 0.0050 |
| S100A14 | 6258.18 | -0.73 | 0.0000 |
| CYBB | 1230.97 | -0.73 | 0.0001 |
| CDC42 | 7462.87 | -0.75 | 0.0000 |
| GMFB | 2390.77 | -0.76 | 0.0000 |
| RORC | 726.62 | -0.76 | 0.0000 |
| TNFRSF1A | 4423.05 | -0.76 | 0.0000 |
| PLAAT4 | 1305.35 | -0.77 | 0.0006 |
| HLA-DPB1 | 4179.55 | -0.77 | 0.0000 |
| TCF7L2 | 2035.90 | -0.77 | 0.0000 |
| CYLD | 1071.68 | -0.78 | 0.0000 |
| HDGFL3 | 541.76 | -0.78 | 0.0001 |
| SEMA3B | 1942.94 | -0.79 | 0.0000 |
| SDC2 | 1324.71 | -0.79 | 0.0000 |
| TRAC | 587.95 | -0.79 | 0.0000 |
| VIM | 10066.42 | -0.80 | 0.0000 |
| CD3D | 191.45 | -0.80 | 0.0000 |
| ISG20 | 802.72 | -0.80 | 0.0000 |
| FCGRT | 9758.17 | -0.80 | 0.0000 |
| IL2RB | 465.89 | -0.81 | 0.0000 |
| HLA-DPA1 | 5355.03 | -0.81 | 0.0000 |
| BMP8B | 472.74 | -0.81 | 0.0000 |
| NFATC1 | 253.58 | -0.83 | 0.0000 |
| HLA-DQA1 | 2230.53 | -0.83 | 0.0000 |
| VCAM1 | 549.98 | -0.83 | 0.0000 |
| PTGER2 | 226.33 | -0.84 | 0.0001 |
| BLNK | 454.07 | -0.84 | 0.0000 |
| RORA | 242.51 | -0.84 | 0.0000 |
| CCN3 | 180.05 | -0.85 | 0.0000 |
| IL18 | 892.18 | -0.86 | 0.0000 |
| FOS | 10817.25 | -0.87 | 0.0000 |
| EGFR | 2367.55 | -0.87 | 0.0000 |
| GBP2 | 2426.37 | -0.87 | 0.0000 |
| CAT | 3651.66 | -0.88 | 0.0000 |
| HCK | 528.43 | -0.89 | 0.0000 |
| HNF4G | 1416.58 | -0.91 | 0.0000 |
| GMFG | 331.16 | -0.91 | 0.0000 |
| ITK | 116.80 | -0.92 | 0.0000 |
| IL1R1 | 1057.10 | -0.92 | 0.0000 |
| MAPK3 | 5252.15 | -0.93 | 0.0000 |
| IGLV3-21 | 1941.61 | -0.93 | 0.0038 |
| HLA-DQA2 | 381.48 | -0.94 | 0.0007 |
| TRBV28 | 104.72 | -0.94 | 0.0000 |
| TGFA | 876.34 | -0.94 | 0.0000 |
| TNFSF13 | 575.72 | -0.95 | 0.0000 |
| VAV1 | 315.29 | -0.95 | 0.0000 |
| HLA-DMB | 1013.79 | -0.96 | 0.0000 |
| C8G | 90.08 | -0.96 | 0.0002 |
| RXRA | 2925.24 | -0.96 | 0.0000 |
| PLA2G2A | 9334.24 | -0.97 | 0.0037 |
| HCST | 76.48 | -0.97 | 0.0000 |
| CTF1 | 61.37 | -0.97 | 0.0000 |
| BCL10 | 1404.10 | -0.98 | 0.0000 |
| IGLV9-49 | 93.75 | -0.98 | 0.0102 |
| PIK3R5 | 191.55 | -0.99 | 0.0000 |
| CD14 | 1936.20 | -0.99 | 0.0000 |
| PPARD | 2225.18 | -1.00 | 0.0000 |
| IGKV1D-13 | 59.88 | -1.01 | 0.0125 |
| CSF1 | 853.07 | -1.01 | 0.0000 |
| CD247 | 157.08 | -1.01 | 0.0000 |
| GRAP2 | 92.76 | -1.02 | 0.0000 |
| LTB | 261.51 | -1.03 | 0.0000 |
| CD4 | 1229.13 | -1.03 | 0.0000 |
| CD28 | 72.50 | -1.04 | 0.0000 |
| SEMA4G | 2319.65 | -1.04 | 0.0000 |
| CD3E | 398.40 | -1.04 | 0.0000 |
| PIK3CD | 360.95 | -1.05 | 0.0000 |
| RETNLB | 671.36 | -1.07 | 0.0023 |
| TNFRSF11A | 1431.35 | -1.07 | 0.0000 |
| TLR8 | 80.75 | -1.08 | 0.0000 |
| B2M | 87109.53 | -1.08 | 0.0000 |
| CD8A | 266.35 | -1.09 | 0.0000 |
| CD3G | 102.90 | -1.09 | 0.0000 |
| IL10RB | 1813.22 | -1.09 | 0.0000 |
| ARRB1 | 1349.72 | -1.09 | 0.0000 |
| NEO1 | 4470.77 | -1.09 | 0.0000 |
| VDR | 3794.23 | -1.10 | 0.0000 |
| SOS2 | 1241.91 | -1.11 | 0.0000 |
| IGHV1-24 | 308.65 | -1.11 | 0.0010 |
| TLR1 | 121.13 | -1.11 | 0.0000 |
| PLXNA2 | 2210.75 | -1.13 | 0.0000 |
| S1PR1 | 416.28 | -1.13 | 0.0000 |
| RBP5 | 103.64 | -1.14 | 0.0000 |
| FAM3D | 9117.16 | -1.14 | 0.0000 |
| IGHV4-61 | 164.84 | -1.16 | 0.0009 |
| IL11RA | 231.87 | -1.17 | 0.0000 |
| PTGFR | 65.08 | -1.17 | 0.0000 |
| SFTPA2 | 84.01 | -1.18 | 0.0001 |
| HLA-DOB | 61.94 | -1.18 | 0.0000 |
| NRP2 | 1259.55 | -1.18 | 0.0000 |
| IGHV1-69 | 208.83 | -1.19 | 0.0006 |
| CTSS | 8885.62 | -1.19 | 0.0000 |
| ITGAL | 456.95 | -1.19 | 0.0000 |
| NDRG1 | 14063.82 | -1.19 | 0.0000 |
| AKT3 | 442.46 | -1.22 | 0.0000 |
| PTPRC | 1039.39 | -1.23 | 0.0000 |
| BTC | 289.10 | -1.23 | 0.0000 |
| FCGR2B | 131.99 | -1.24 | 0.0000 |
| PDGFRA | 1147.75 | -1.24 | 0.0000 |
| PTGER3 | 142.66 | -1.25 | 0.0000 |
| FGFR1 | 1179.37 | -1.25 | 0.0000 |
| NPR3 | 114.98 | -1.25 | 0.0000 |
| MUC4 | 3556.03 | -1.27 | 0.0000 |
| PTK2B | 2006.86 | -1.27 | 0.0000 |
| LTF | 198.01 | -1.27 | 0.0000 |
| EBI3 | 61.93 | -1.28 | 0.0000 |
| KITLG | 963.00 | -1.28 | 0.0000 |
| CSF1R | 1244.25 | -1.28 | 0.0000 |
| CSF2RB | 464.59 | -1.28 | 0.0000 |
| IGLV3-27 | 103.86 | -1.29 | 0.0002 |
| RNASEL | 469.54 | -1.30 | 0.0000 |
| LTBP4 | 5105.17 | -1.30 | 0.0000 |
| LGR4 | 2633.82 | -1.30 | 0.0000 |
| IGHV2-70 | 116.73 | -1.31 | 0.0003 |
| CCL11 | 196.12 | -1.31 | 0.0000 |
| TNFSF12 | 424.83 | -1.32 | 0.0000 |
| F2RL1 | 3473.12 | -1.32 | 0.0000 |
| A2M | 10289.29 | -1.32 | 0.0000 |
| TGFBR3 | 754.61 | -1.32 | 0.0000 |
| LEAP2 | 57.56 | -1.33 | 0.0000 |
| CCL5 | 715.00 | -1.33 | 0.0000 |
| IL6ST | 2267.84 | -1.33 | 0.0000 |
| CCR7 | 112.21 | -1.34 | 0.0000 |
| ACVR1C | 226.77 | -1.34 | 0.0000 |
| PTGDS | 646.23 | -1.35 | 0.0000 |
| SSTR1 | 269.48 | -1.35 | 0.0000 |
| FGF13 | 106.89 | -1.36 | 0.0000 |
| ILK | 452.54 | -1.37 | 0.0000 |
| TEK | 226.98 | -1.37 | 0.0000 |
| IGHD | 111.20 | -1.39 | 0.0001 |
| FAS | 600.94 | -1.40 | 0.0000 |
| OASL | 575.92 | -1.40 | 0.0000 |
| IGLV3-25 | 986.27 | -1.40 | 0.0000 |
| EDNRB | 368.49 | -1.41 | 0.0000 |
| IL10RA | 553.09 | -1.42 | 0.0000 |
| CHP1 | 5855.73 | -1.42 | 0.0000 |
| ADA2 | 1053.51 | -1.43 | 0.0000 |
| CMKLR1 | 306.81 | -1.43 | 0.0000 |
| NRG1 | 99.42 | -1.46 | 0.0000 |
| IGKV1D-8 | 73.11 | -1.48 | 0.0000 |
| IGKV3-15 | 885.67 | -1.49 | 0.0000 |
| IGLC2 | 5040.68 | -1.49 | 0.0000 |
| CCL15 | 192.90 | -1.50 | 0.0000 |
| KL | 58.92 | -1.50 | 0.0000 |
| LEPR | 229.70 | -1.51 | 0.0000 |
| TNFSF10 | 2579.53 | -1.52 | 0.0000 |
| ACVRL1 | 2569.65 | -1.52 | 0.0000 |
| VIPR1 | 1497.58 | -1.54 | 0.0000 |
| FGF7 | 211.83 | -1.54 | 0.0000 |
| GNAI1 | 487.06 | -1.55 | 0.0000 |
| IGHV3-30 | 781.15 | -1.56 | 0.0000 |
| PTGER4 | 974.72 | -1.57 | 0.0000 |
| HMOX1 | 1121.27 | -1.59 | 0.0000 |
| RSAD2 | 417.48 | -1.59 | 0.0000 |
| IGHV3-20 | 54.40 | -1.60 | 0.0000 |
| BTK | 144.26 | -1.61 | 0.0000 |
| IGHV3-33 | 520.45 | -1.61 | 0.0000 |
| HSPA2 | 1078.82 | -1.63 | 0.0000 |
| ANGPT1 | 97.72 | -1.63 | 0.0000 |
| IGHV4-34 | 496.33 | -1.65 | 0.0000 |
| BACH2 | 60.21 | -1.65 | 0.0000 |
| PDGFD | 211.21 | -1.69 | 0.0000 |
| CD1D | 101.66 | -1.69 | 0.0000 |
| IGLV3-19 | 1501.61 | -1.70 | 0.0000 |
| IGHV4-31 | 326.84 | -1.70 | 0.0000 |
| IGLV1-36 | 96.97 | -1.70 | 0.0000 |
| PI15 | 134.64 | -1.72 | 0.0000 |
| IGHV2-26 | 167.80 | -1.73 | 0.0000 |
| PTGDR | 148.70 | -1.73 | 0.0000 |
| IGHV1-46 | 754.76 | -1.73 | 0.0000 |
| NPR1 | 168.74 | -1.74 | 0.0000 |
| IGHV3-48 | 255.53 | -1.74 | 0.0000 |
| IGLV1-40 | 1476.48 | -1.75 | 0.0000 |
| SLC22A17 | 211.75 | -1.75 | 0.0000 |
| IGHV4-28 | 149.38 | -1.76 | 0.0000 |
| IGHM | 9717.16 | -1.77 | 0.0000 |
| IGLV2-11 | 1274.61 | -1.77 | 0.0000 |
| AHNAK | 18508.78 | -1.79 | 0.0000 |
| IGKV1-5 | 2809.95 | -1.80 | 0.0000 |
| TNFRSF13C | 84.74 | -1.81 | 0.0000 |
| IGLV6-57 | 704.61 | -1.81 | 0.0000 |
| UCN3 | 53.76 | -1.83 | 0.0000 |
| IGLC3 | 3009.96 | -1.83 | 0.0000 |
| COLEC12 | 270.92 | -1.83 | 0.0000 |
| NR4A3 | 200.90 | -1.83 | 0.0000 |
| IL16 | 321.06 | -1.83 | 0.0000 |
| IGLV1-47 | 711.02 | -1.84 | 0.0000 |
| IGHV4-39 | 1477.35 | -1.85 | 0.0000 |
| NGFR | 121.77 | -1.85 | 0.0000 |
| FGF2 | 144.49 | -1.85 | 0.0000 |
| IGHV3-43 | 102.57 | -1.85 | 0.0000 |
| CYSLTR1 | 58.32 | -1.85 | 0.0000 |
| IGLC7 | 143.78 | -1.86 | 0.0000 |
| PLCG2 | 412.09 | -1.86 | 0.0000 |
| IGHV1-18 | 1236.92 | -1.87 | 0.0000 |
| CD48 | 329.98 | -1.87 | 0.0000 |
| TLR7 | 76.16 | -1.88 | 0.0000 |
| SPINK5 | 215.74 | -1.89 | 0.0000 |
| PIK3CG | 118.59 | -1.89 | 0.0000 |
| IGLV2-23 | 1648.04 | -1.89 | 0.0000 |
| FABP4 | 202.45 | -1.90 | 0.0000 |
| IGLV1-51 | 1146.15 | -1.93 | 0.0000 |
| BMP2 | 710.84 | -1.93 | 0.0000 |
| BMP6 | 78.63 | -1.93 | 0.0000 |
| NR3C1 | 556.73 | -1.93 | 0.0000 |
| IGKV2D-29 | 144.68 | -1.94 | 0.0000 |
| DEFB1 | 137.14 | -1.94 | 0.0000 |
| IGKV3D-11 | 66.40 | -1.95 | 0.0000 |
| SEMA3G | 233.67 | -1.95 | 0.0000 |
| CXCL13 | 246.92 | -1.95 | 0.0000 |
| AR | 73.97 | -1.96 | 0.0000 |
| IGHV3-53 | 260.79 | -1.96 | 0.0000 |
| IGLV3-10 | 482.42 | -1.97 | 0.0000 |
| IGKV1-6 | 363.31 | -1.99 | 0.0000 |
| ADIPOQ | 93.00 | -2.01 | 0.0183 |
| IGKV1-8 | 91.38 | -2.03 | 0.0000 |
| SLIT2 | 221.25 | -2.03 | 0.0000 |
| IGKV4-1 | 3619.14 | -2.03 | 0.0000 |
| IGHV3-64 | 78.16 | -2.04 | 0.0000 |
| IGLV5-45 | 170.70 | -2.05 | 0.0000 |
| IGHV3-23 | 1963.50 | -2.07 | 0.0000 |
| IGLV4-69 | 612.40 | -2.07 | 0.0000 |
| IL1R2 | 479.79 | -2.07 | 0.0000 |
| TLR3 | 393.20 | -2.08 | 0.0000 |
| IGKC | 40182.35 | -2.08 | 0.0000 |
| IGKV3-11 | 2924.57 | -2.09 | 0.0000 |
| IGHV3-73 | 276.86 | -2.09 | 0.0000 |
| IGLV2-18 | 143.49 | -2.09 | 0.0000 |
| IGLV2-14 | 1869.53 | -2.10 | 0.0000 |
| GPER1 | 125.83 | -2.10 | 0.0000 |
| CD19 | 61.64 | -2.11 | 0.0000 |
| IGKV1D-16 | 89.70 | -2.12 | 0.0000 |
| IGLV7-46 | 438.78 | -2.14 | 0.0000 |
| CCL28 | 1211.37 | -2.14 | 0.0000 |
| IGLV3-9 | 232.54 | -2.15 | 0.0000 |
| IGHV5-51 | 1523.76 | -2.15 | 0.0000 |
| IGHV3-15 | 1067.22 | -2.16 | 0.0000 |
| IGHV3-66 | 110.88 | -2.17 | 0.0000 |
| ACKR1 | 297.54 | -2.19 | 0.0000 |
| IGKV2-24 | 334.41 | -2.19 | 0.0000 |
| CCL21 | 686.77 | -2.20 | 0.0000 |
| IGKV1-9 | 647.40 | -2.22 | 0.0000 |
| IGLV1-44 | 845.45 | -2.23 | 0.0000 |
| IGLV4-60 | 135.12 | -2.24 | 0.0000 |
| CD79B | 128.94 | -2.25 | 0.0000 |
| IGHV3-13 | 126.43 | -2.26 | 0.0000 |
| IGHV4-59 | 924.93 | -2.26 | 0.0000 |
| IGLV3-1 | 596.20 | -2.28 | 0.0000 |
| IGKV1-27 | 348.76 | -2.29 | 0.0000 |
| NR5A2 | 488.91 | -2.30 | 0.0000 |
| FGFR2 | 681.42 | -2.30 | 0.0000 |
| IGHV3-49 | 496.33 | -2.31 | 0.0000 |
| IGKV1-16 | 444.44 | -2.32 | 0.0000 |
| CD209 | 264.52 | -2.32 | 0.0000 |
| CSRP1 | 10438.97 | -2.33 | 0.0000 |
| SEMA3D | 54.13 | -2.34 | 0.0000 |
| IGLV8-61 | 437.67 | -2.34 | 0.0000 |
| CCL19 | 161.09 | -2.34 | 0.0000 |
| IGKV3-20 | 2991.83 | -2.34 | 0.0000 |
| SCG2 | 151.86 | -2.36 | 0.0000 |
| SEMA6A | 1060.87 | -2.38 | 0.0000 |
| IGHV3-11 | 603.22 | -2.38 | 0.0000 |
| IGHV3-21 | 587.33 | -2.39 | 0.0000 |
| CCL8 | 73.67 | -2.39 | 0.0000 |
| CCL13 | 105.30 | -2.39 | 0.0000 |
| MAPT | 80.97 | -2.41 | 0.0000 |
| GHR | 135.27 | -2.42 | 0.0000 |
| IGKV3D-20 | 172.48 | -2.43 | 0.0000 |
| IGHV1-58 | 101.75 | -2.43 | 0.0000 |
| PTGDR2 | 79.75 | -2.44 | 0.0000 |
| IGKV1-17 | 436.32 | -2.44 | 0.0000 |
| THRB | 331.30 | -2.45 | 0.0000 |
| CD79A | 405.93 | -2.47 | 0.0000 |
| IGHA1 | 55666.66 | -2.49 | 0.0000 |
| IGHV1-2 | 579.59 | -2.50 | 0.0000 |
| S100B | 120.59 | -2.51 | 0.0000 |
| PRKCB | 255.81 | -2.53 | 0.0000 |
| IGKV3-7 | 52.45 | -2.56 | 0.0000 |
| CHGB | 141.33 | -2.56 | 0.0000 |
| IGHV6-1 | 51.56 | -2.58 | 0.0000 |
| BMP5 | 93.55 | -2.59 | 0.0000 |
| IGHV3-74 | 814.63 | -2.60 | 0.0000 |
| CD22 | 133.94 | -2.60 | 0.0000 |
| IGLV2-8 | 589.62 | -2.64 | 0.0000 |
| TPM2 | 6720.67 | -2.69 | 0.0000 |
| IGKV6-21 | 75.34 | -2.73 | 0.0000 |
| EDN2 | 51.80 | -2.74 | 0.0000 |
| IGLV7-43 | 235.91 | -2.77 | 0.0000 |
| CXCL12 | 1091.50 | -2.77 | 0.0000 |
| IL6R | 603.06 | -2.78 | 0.0000 |
| NR3C2 | 979.90 | -2.78 | 0.0000 |
| PTN | 124.19 | -2.79 | 0.0000 |
| IGLV10-54 | 266.13 | -2.80 | 0.0000 |
| IGHV3-72 | 316.00 | -2.89 | 0.0000 |
| SECTM1 | 1290.19 | -2.89 | 0.0000 |
| CR2 | 207.67 | -2.92 | 0.0000 |
| IGHV2-5 | 97.92 | -2.96 | 0.0000 |
| SEMA6D | 369.15 | -3.03 | 0.0000 |
| FABP2 | 419.79 | -3.15 | 0.0000 |
| EDN3 | 408.85 | -3.20 | 0.0000 |
| TNFRSF17 | 81.08 | -3.28 | 0.0000 |
| CTSG | 50.31 | -3.32 | 0.0000 |
| VIP | 320.30 | -3.34 | 0.0000 |
| MASP1 | 215.05 | -3.38 | 0.0000 |
| IGHA2 | 37633.30 | -3.41 | 0.0000 |
| LIFR | 172.61 | -3.46 | 0.0000 |
| CNTFR | 98.27 | -3.69 | 0.0000 |
| IGKV2-30 | 54.93 | -3.72 | 0.0000 |
| DES | 12232.59 | -3.74 | 0.0000 |
| GREM2 | 265.41 | -3.76 | 0.0000 |
| NR1H4 | 73.52 | -3.88 | 0.0000 |
| CHP2 | 1710.76 | -3.90 | 0.0000 |
| IGHV3-7 | 50.92 | -3.93 | 0.0000 |
| GLP2R | 63.75 | -3.95 | 0.0000 |
| OGN | 220.47 | -4.18 | 0.0000 |
| ANGPTL1 | 126.03 | -4.34 | 0.0000 |
| CHGA | 1217.02 | -4.48 | 0.0000 |
| GCG | 263.28 | -4.70 | 0.0000 |
| GUCA2A | 2141.09 | -5.06 | 0.0000 |
| BMP3 | 235.01 | -5.22 | 0.0000 |
| SST | 73.48 | -5.32 | 0.0000 |
| PYY | 328.52 | -5.59 | 0.0000 |
| INSL5 | 175.30 | -5.77 | 0.0000 |

**Additional file 1: Table S2 The screened pairs of DMPs and IR-DEGs by Spearman correlation analysis**

| **IR-DEGs** | **DMPs** | **P value** | **R value** |
| --- | --- | --- | --- |
| IL23A | cg00294382 | 0.000 | -0.609 |
| PTGFR | cg00701741 | 0.000 | -0.382 |
| BST2 | cg01329005 | 0.000 | -0.722 |
| HSPA1A | cg01639032 | 0.000 | -0.710 |
| BMP4 | cg01873886 | 0.000 | -0.366 |
| PDGFD | cg02120582 | 0.000 | -0.306 |
| BMP3 | cg02621694 | 0.000 | -0.317 |
| PI3 | cg02733351 | 0.000 | -0.338 |
| INPP5D | cg02935132 | 0.000 | -0.736 |
| PTGFR | cg03495868 | 0.000 | -0.444 |
| LIFR | cg03723506 | 0.000 | -0.312 |
| PDGFRA | cg03792767 | 0.000 | -0.345 |
| PDGFRA | cg03966785 | 0.000 | -0.302 |
| PGC | cg03991152 | 0.000 | -0.610 |
| INPP5D | cg04126261 | 0.000 | -0.609 |
| CD1D | cg04360049 | 0.000 | -0.331 |
| BMP4 | cg04937184 | 0.000 | -0.409 |
| IFITM1 | cg05432003 | 0.000 | -0.555 |
| LIFR | cg05923785 | 0.000 | -0.379 |
| INPP5D | cg06272010 | 0.000 | -0.728 |
| SERPIND1 | cg06658625 | 0.000 | -0.672 |
| CD8A | cg06804210 | 0.000 | -0.413 |
| INPP5D | cg07178563 | 0.000 | -0.761 |
| PDGFRA | cg08038052 | 0.000 | -0.360 |
| BMP4 | cg08046044 | 0.000 | -0.390 |
| DEFA6 | cg08312330 | 0.000 | -0.373 |
| LIFR | cg08392199 | 0.000 | -0.433 |
| SLIT2 | cg08428452 | 0.000 | -0.395 |
| IFITM1 | cg09026253 | 0.000 | -0.614 |
| HSPA1A | cg09085842 | 0.000 | -0.683 |
| SLC22A17 | cg09321747 | 0.000 | -0.345 |
| CCL20 | cg09425228 | 0.000 | -0.335 |
| SLIT2 | cg09781944 | 0.000 | -0.370 |
| BST2 | cg09993699 | 0.000 | -0.555 |
| SLC22A17 | cg10058779 | 0.000 | -0.401 |
| AZGP1 | cg10372302 | 0.000 | -0.411 |
| PDGFRA | cg10492953 | 0.000 | -0.342 |
| IFITM1 | cg10552523 | 0.000 | -0.595 |
| BST2 | cg11558551 | 0.000 | -0.646 |
| EREG | cg11646192 | 0.000 | -0.703 |
| IFITM1 | cg11694510 | 0.000 | -0.648 |
| LIFR | cg11841722 | 0.000 | -0.316 |
| NFATC1 | cg11928275 | 0.000 | -0.393 |
| AZGP1 | cg12019109 | 0.000 | -0.446 |
| CD1D | cg12124922 | 0.000 | -0.329 |
| LIFR | cg12602374 | 0.000 | -0.331 |
| PGC | cg12855851 | 0.000 | -0.562 |
| HSPA1A | cg13024590 | 0.000 | -0.632 |
| SLIT2 | cg13078140 | 0.000 | -0.487 |
| SST | cg13206017 | 0.000 | -0.326 |
| CNTFR | cg13226172 | 0.000 | -0.367 |
| SLIT2 | cg13281139 | 0.000 | -0.467 |
| NR3C1 | cg13648501 | 0.000 | -0.414 |
| S100P | cg14140379 | 0.000 | -0.531 |
| S100P | cg14323984 | 0.000 | -0.575 |
| SDC2 | cg14538332 | 0.000 | -0.336 |
| NR3C1 | cg14558428 | 0.000 | -0.300 |
| PDGFRA | cg14777772 | 0.000 | -0.366 |
| S100P | cg14900031 | 0.000 | -0.531 |
| INPP5D | cg15131282 | 0.000 | -0.691 |
| HSPA1A | cg15185479 | 0.000 | -0.699 |
| SLIT2 | cg15469350 | 0.000 | -0.445 |
| SLC22A17 | cg15476528 | 0.000 | -0.325 |
| INPP5D | cg16321975 | 0.000 | -0.755 |
| BMP2 | cg16831623 | 0.000 | -0.301 |
| IL23A | cg16865138 | 0.000 | -0.566 |
| S1PR1 | cg17859448 | 0.000 | -0.375 |
| RORC | cg18149207 | 0.000 | -0.641 |
| LIFR | cg18174928 | 0.000 | -0.321 |
| PDGFD | cg18403606 | 0.000 | -0.326 |
| CCRL2 | cg18599081 | 0.000 | -0.303 |
| HSPA1A | cg18846140 | 0.000 | -0.701 |
| LIFR | cg18848688 | 0.000 | -0.319 |
| SLIT2 | cg18972811 | 0.000 | -0.435 |
| EREG | cg19308222 | 0.000 | -0.747 |
| BMP2 | cg19528338 | 0.000 | -0.303 |
| S100A11 | cg19930352 | 0.000 | -0.430 |
| IL23A | cg19951006 | 0.000 | -0.535 |
| BST2 | cg20092122 | 0.000 | -0.751 |
| RBP1 | cg20532370 | 0.000 | -0.658 |
| IFITM1 | cg20566897 | 0.000 | -0.687 |
| BMP3 | cg20631104 | 0.000 | -0.310 |
| HSPA1A | cg21122656 | 0.000 | -0.722 |
| NR3C1 | cg21702128 | 0.000 | -0.303 |
| INPP5D | cg22029587 | 0.000 | -0.669 |
| PDGFRA | cg23209990 | 0.000 | -0.401 |
| NR3C2 | cg23329208 | 0.000 | -0.378 |
| CCRL2 | cg23350385 | 0.000 | -0.303 |
| TRPC4AP | cg23414001 | 0.000 | -0.491 |
| SLC22A17 | cg23464698 | 0.000 | -0.334 |
| MET | cg24258705 | 0.000 | -0.529 |
| BMP4 | cg24526899 | 0.000 | -0.547 |
| IL23A | cg24773560 | 0.000 | -0.566 |
| GHR | cg24773720 | 0.000 | -0.332 |
| PIK3R5 | cg26239391 | 0.000 | -0.325 |
| AZGP1 | cg26680675 | 0.000 | -0.585 |
| BMP3 | cg26917673 | 0.000 | -0.300 |
| VIM | cg26983469 | 0.000 | -0.343 |
| S100P | cg27027375 | 0.000 | -0.488 |
| VIM | cg27313572 | 0.000 | -0.394 |
| RBP1 | cg27457941 | 0.000 | -0.609 |
| CCL8 | cg00038857 | 0.000 | 0.498 |
| PI15 | cg00128482 | 0.000 | 0.369 |
| GHR | cg00394261 | 0.000 | 0.541 |
| HLA-DOA | cg00988577 | 0.000 | 0.403 |
| MAPT | cg01640727 | 0.000 | 0.366 |
| IL1RN | cg01991967 | 0.000 | 0.340 |
| LIFR | cg04444661 | 0.000 | 0.434 |
| CCL8 | cg04656009 | 0.000 | 0.369 |
| RASGRP3 | cg04707603 | 0.000 | 0.555 |
| IL16 | cg05042034 | 0.000 | 0.354 |
| HLA-DOA | cg05043389 | 0.000 | 0.319 |
| CCL13 | cg05281206 | 0.000 | 0.511 |
| CCL8 | cg06083483 | 0.000 | 0.479 |
| CARD11 | cg06173509 | 0.000 | 0.579 |
| CD86 | cg06327732 | 0.000 | 0.449 |
| RARB | cg06705767 | 0.000 | 0.334 |
| NR3C1 | cg07589972 | 0.000 | 0.452 |
| IL16 | cg08255105 | 0.000 | 0.369 |
| CCL11 | cg08499840 | 0.000 | 0.342 |
| BACH2 | cg11559054 | 0.000 | 0.443 |
| CARD11 | cg14168009 | 0.000 | 0.703 |
| CARD11 | cg15638239 | 0.000 | 0.658 |
| NR1H4 | cg15990724 | 0.000 | 0.330 |
| BACH2 | cg18477569 | 0.000 | 0.528 |
| NR1H4 | cg18944984 | 0.000 | 0.375 |
| CCL2 | cg21109025 | 0.000 | 0.340 |
| RASGRP3 | cg21244038 | 0.000 | 0.367 |
| SCG2 | cg21269859 | 0.000 | 0.357 |
| BACH2 | cg23244234 | 0.000 | 0.422 |
| CARD11 | cg23352157 | 0.000 | 0.642 |
| CD1D | cg24432768 | 0.000 | 0.454 |
| HLA-DOA | cg24476363 | 0.000 | 0.411 |
| BACH2 | cg24700316 | 0.000 | 0.541 |
| LEPR | cg26342890 | 0.000 | 0.372 |
| CCL8 | cg27000831 | 0.000 | 0.493 |
| BACH2 | cg27277366 | 0.000 | 0.552 |

**Additional file 1: Table S3 Tumor-infiltrating immune cells correlated with the risk between normal and cancer**

| **Cell_type** | **Normal (n=51)** | | **Cancer (622)** | |
| --- | --- | --- | --- | --- |
|  | **Mean** | **Std** | **Mean** | **Std** |
| Mast cell activated_CIBERSORT | 0.08 | 0.04 | 0.01 | 0.02 |
| B cell plasma_CIBERSORT | 0.13 | 0.07 | 0.05 | 0.06 |
| Macrophage M0_CIBERSORT | 0.00 | 0.01 | 0.14 | 0.12 |
| Monocyte_CIBERSORT | 0.03 | 0.03 | 0.01 | 0.02 |
| Mast cell resting_CIBERSORT | 0.01 | 0.04 | 0.08 | 0.07 |
| Macrophage M2_CIBERSORT | 0.28 | 0.08 | 0.21 | 0.09 |
| T cell follicular helper_CIBERSORT | 0.03 | 0.02 | 0.06 | 0.04 |
| Macrophage M1_CIBERSORT | 0.03 | 0.05 | 0.06 | 0.04 |
| NK cell resting_CIBERSORT | 0.00 | 0.01 | 0.02 | 0.03 |
| T cell CD4+ memory resting_CIBERSORT | 0.19 | 0.05 | 0.15 | 0.08 |
| T cell regulatory (Tregs)_CIBERSORT | 0.02 | 0.02 | 0.04 | 0.03 |
| B cell naive_CIBERSORT | 0.04 | 0.06 | 0.02 | 0.04 |
| T cell CD4+ memory activated_CIBERSORT | 0.00 | 0.00 | 0.02 | 0.03 |
| B cell memory_CIBERSORT | 0.01 | 0.01 | 0.00 | 0.01 |
| Neutrophil_CIBERSORT | 0.00 | 0.01 | 0.01 | 0.02 |
| Mast cell activated_CIBERSORT-ABS | 0.05 | 0.03 | 0.00 | 0.01 |
| B cell plasma_CIBERSORT-ABS | 0.08 | 0.05 | 0.02 | 0.02 |
| T cell CD4+ memory resting_CIBERSORT-ABS | 0.13 | 0.05 | 0.06 | 0.04 |
| Monocyte_CIBERSORT-ABS | 0.02 | 0.02 | 0.00 | 0.01 |
| Macrophage M2_CIBERSORT-ABS | 0.18 | 0.06 | 0.09 | 0.07 |
| B cell naive_CIBERSORT-ABS | 0.03 | 0.06 | 0.01 | 0.02 |
| Macrophage M0_CIBERSORT-ABS | 0.00 | 0.01 | 0.05 | 0.06 |
| NK cell activated_CIBERSORT-ABS | 0.02 | 0.01 | 0.01 | 0.01 |
| T cell CD8+_CIBERSORT-ABS | 0.07 | 0.05 | 0.04 | 0.04 |
| B cell memory_CIBERSORT-ABS | 0.01 | 0.01 | 0.00 | 0.01 |
| Mast cell resting_CIBERSORT-ABS | 0.01 | 0.03 | 0.03 | 0.03 |
| Myeloid dendritic cell resting_CIBERSORT-ABS | 0.01 | 0.01 | 0.00 | 0.01 |
| NK cell resting_CIBERSORT-ABS | 0.00 | 0.00 | 0.01 | 0.01 |
| T cell CD4+ memory activated_CIBERSORT-ABS | 0.00 | 0.00 | 0.01 | 0.01 |
| Eosinophil_CIBERSORT-ABS | 0.00 | 0.00 | 0.00 | 0.00 |
| Neutrophil_CIBERSORT-ABS | 0.00 | 0.01 | 0.01 | 0.01 |
| B cell_EPIC | 0.02 | 0.02 | 0.00 | 0.01 |
| T cell CD4+_EPIC | 0.05 | 0.01 | 0.03 | 0.01 |
| Endothelial cell_EPIC | 0.03 | 0.01 | 0.02 | 0.01 |
| T cell CD8+_EPIC | 0.02 | 0.01 | 0.01 | 0.01 |
| Macrophage_EPIC | 0.01 | 0.01 | 0.01 | 0.01 |
| Cancer associated fibroblast_EPIC | 0.02 | 0.01 | 0.06 | 0.08 |
| Neutrophil_MCPCOUNTER | 44.23 | 23.41 | 6.39 | 3.73 |
| Myeloid dendritic cell_MCPCOUNTER | 5.17 | 2.31 | 1.46 | 1.47 |
| B cell_MCPCOUNTER | 12.90 | 13.15 | 2.45 | 4.96 |
| Endothelial cell_MCPCOUNTER | 8.93 | 2.66 | 5.84 | 3.35 |
| NK cell_MCPCOUNTER | 1.35 | 0.57 | 0.40 | 1.16 |
| T cell_MCPCOUNTER | 5.15 | 1.93 | 3.69 | 2.07 |
| Cancer associated fibroblast_MCPCOUNTER | 570.08 | 818.01 | 295.47 | 347.44 |
| Monocyte_MCPCOUNTER | 10.97 | 4.22 | 7.34 | 5.79 |
| Macrophage/Monocyte_MCPCOUNTER | 10.97 | 4.22 | 7.34 | 5.79 |
| uncharacterized cell_QUANTISEQ | 0.58 | 0.09 | 0.81 | 0.06 |
| Neutrophil_QUANTISEQ | 0.15 | 0.05 | 0.06 | 0.02 |
| T cell CD4+ (non-regulatory)_QUANTISEQ | 0.07 | 0.03 | 0.02 | 0.02 |
| B cell_QUANTISEQ | 0.02 | 0.02 | 0.01 | 0.01 |
| Macrophage M1_QUANTISEQ | 0.09 | 0.03 | 0.05 | 0.03 |
| T cell regulatory (Tregs)_QUANTISEQ | 0.02 | 0.01 | 0.01 | 0.01 |
| Macrophage M2_QUANTISEQ | 0.04 | 0.05 | 0.02 | 0.01 |
| NK cell_QUANTISEQ | 0.02 | 0.01 | 0.01 | 0.01 |
| Myeloid dendritic cell_QUANTISEQ | 0.00 | 0.00 | 0.00 | 0.00 |
| T cell CD8+_TIMER | 0.28 | 0.07 | 0.16 | 0.08 |
| B cell_TIMER | 0.17 | 0.12 | 0.08 | 0.06 |
| Macrophage_TIMER | 0.11 | 0.07 | 0.05 | 0.06 |
| Myeloid dendritic cell_TIMER | 0.68 | 0.10 | 0.49 | 0.18 |
| T cell CD4+_TIMER | 0.18 | 0.07 | 0.14 | 0.06 |
| Neutrophil_TIMER | 0.14 | 0.03 | 0.12 | 0.06 |
| Class-switched memory B cell_XCELL | 0.07 | 0.04 | 0.01 | 0.02 |
| B cell memory_XCELL | 0.07 | 0.06 | 0.01 | 0.02 |
| microenvironment score_XCELL | 0.25 | 0.10 | 0.07 | 0.08 |
| B cell_XCELL | 0.14 | 0.14 | 0.02 | 0.05 |
| immune score_XCELL | 0.19 | 0.11 | 0.06 | 0.07 |
| B cell plasma_XCELL | 0.03 | 0.02 | 0.01 | 0.01 |
| Plasmacytoid dendritic cell_XCELL | 0.05 | 0.03 | 0.01 | 0.02 |
| Cancer associated fibroblast_XCELL | 0.08 | 0.11 | 0.01 | 0.03 |
| T cell NK_XCELL | 0.12 | 0.06 | 0.06 | 0.04 |
| Macrophage M1_XCELL | 0.03 | 0.01 | 0.01 | 0.02 |
| stroma score_XCELL | 0.06 | 0.07 | 0.01 | 0.03 |
| T cell CD4+ Th1_XCELL | 0.00 | 0.01 | 0.06 | 0.04 |
| Myeloid dendritic cell_XCELL | 0.03 | 0.02 | 0.01 | 0.02 |
| Myeloid dendritic cell activated_XCELL | 0.29 | 0.09 | 0.15 | 0.11 |
| Hematopoietic stem cell_XCELL | 0.15 | 0.07 | 0.07 | 0.06 |
| Mast cell_XCELL | 0.01 | 0.01 | 0.01 | 0.00 |
| Common myeloid progenitor_XCELL | 0.00 | 0.00 | 0.00 | 0.00 |
| Eosinophil_XCELL | 0.01 | 0.01 | 0.00 | 0.01 |
| B cell naive_XCELL | 0.01 | 0.03 | 0.00 | 0.01 |
| Granulocyte-monocyte progenitor_XCELL | 0.03 | 0.03 | 0.01 | 0.02 |
| Common lymphoid progenitor_XCELL | 0.06 | 0.02 | 0.09 | 0.03 |
| T cell CD8+ central memory_XCELL | 0.04 | 0.03 | 0.02 | 0.03 |
| T cell CD4+ Th2_XCELL | 0.03 | 0.04 | 0.08 | 0.06 |
| Neutrophil_XCELL | 0.01 | 0.01 | 0.00 | 0.01 |
| Monocyte_XCELL | 0.03 | 0.03 | 0.01 | 0.03 |
| T cell CD8+ naive_XCELL | 0.01 | 0.01 | 0.01 | 0.01 |
| Macrophage_XCELL | 0.02 | 0.01 | 0.01 | 0.02 |
| Endothelial cell_XCELL | 0.02 | 0.02 | 0.01 | 0.02 |
| T cell regulatory (Tregs)_XCELL | 0.00 | 0.00 | 0.00 | 0.01 |
| T cell gamma delta_XCELL | 0.00 | 0.00 | 0.00 | 0.00 |
| T cell CD4+ central memory_XCELL | 0.00 | 0.00 | 0.01 | 0.01 |
| T cell CD8+_XCELL | 0.02 | 0.03 | 0.01 | 0.02 |

**
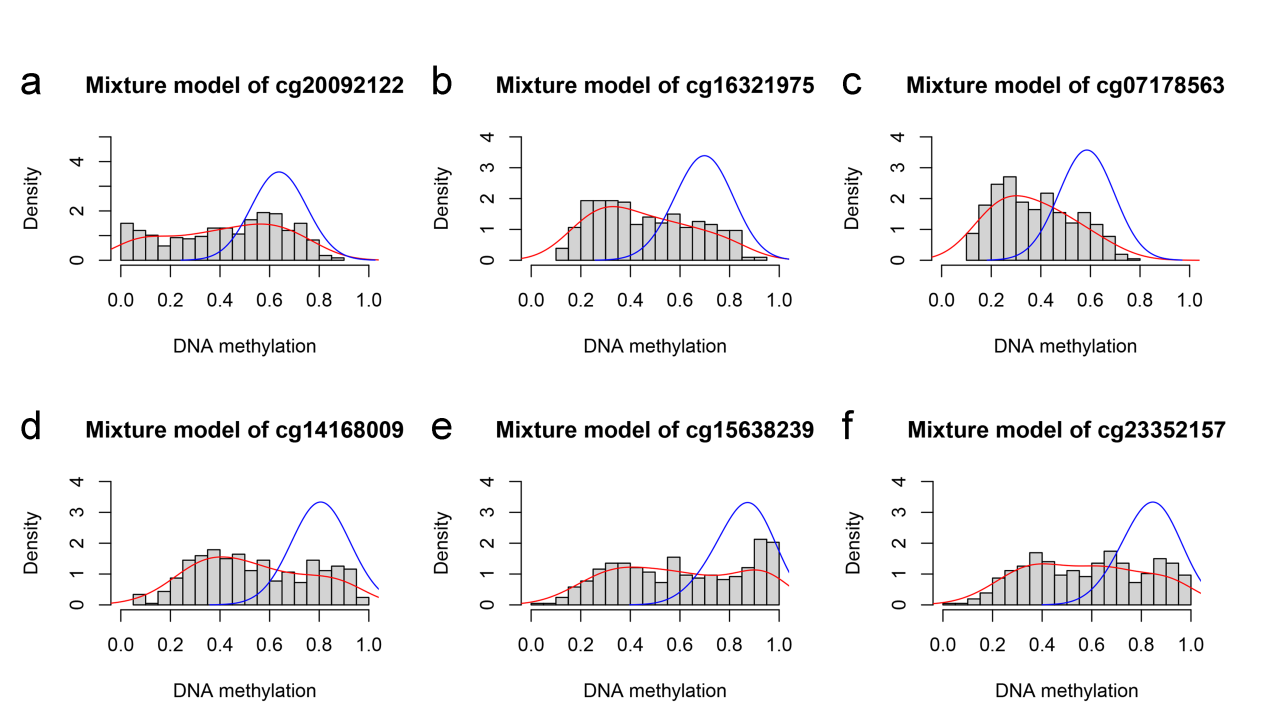
**

**Additional file 1: Figure S1. Methylation distributions for the top three negative (a-c)/positive (d-f) correlations of IR-DEGs.**

The abscissa is the degree of methylation, the ordinate is the density of methylated samples, the histogram represents the methylation distribution of the tumor samples, and the curve demonstrates the simulated trend curve of the methylation distribution in the tumor samples. The red line represents the distribution of methylation in tumor samples. The blue line represents the distribution of methylation in control samples.
